# Coming together to define membrane contact sites

**DOI:** 10.1038/s41467-019-09253-3

**Published:** 2019-03-20

**Authors:** Luca Scorrano, Maria Antonietta De Matteis, Scott Emr, Francesca Giordano, György Hajnóczky, Benoît Kornmann, Laura L. Lackner, Tim P. Levine, Luca Pellegrini, Karin Reinisch, Rosario Rizzuto, Thomas Simmen, Harald Stenmark, Christian Ungermann, Maya Schuldiner

**Affiliations:** 1 Venetian Institute of Molecular Medicine, Department of Biology, University of Padua, Padua, Italy; 2 Telethon Institute of Genetics and Medicine, Pozzuoli, Naples Italy; 3 Department of Molecular Medicine and Medical Biotechnology, University of Napoli Federico II, Naples, Italy; 4 Weill Institute for Cell and Molecular Biology and Department of Molecular Biology and Genetics, Cornell University, Ithaca, NY USA; 5 Institute for Integrative Biology of the Cell (I2BC), CEA, CNRS, Paris-Sud University, Paris-Saclay University, Gif-sur-Yvette cedex, 91198 France; 6 MitoCare Center, Department of Pathology, Anatomy and Cell Biology, Thomas Jefferson University, Philadelphia, PA USA; 7 University of Oxford, Department of Biochemistry, South Parks Road, Ox1 3QU Oxford, United Kingdom; 8 Department of Molecular Biosciences, Northwestern University, Evanston, IL 60208 USA; 9 UCL Institute of Ophthalmology, London, EC1V 9EL UK; 10 Department of Molecular Biology, Medical Biochemistry, and Pathology, Universitè Laval, Quebec, QC Canada; 11 Department of Cell Biology, Yale University School of Medicine, New Haven, CT 06520 USA; 12 Department of Biomedical Sciences, University of Padua, Padua, Italy; 13 University of Alberta, Faculty of Medicine and Dentistry, Department of Cell Biology, Edmonton, AB T6G2H7 Canada; 14 Centre for Cancer Cell Reprogramming, Faculty of Medicine, University of Oslo, Montebello, N-0379 Oslo, Norway; 15 Department of Biology/Chemistry, University of Osnabrück, 49082 Osnabrück, Germany; 16 Department of Molecular Genetics, Weizmann Institute of Science, Rehovot, 7610001 Israel

## Abstract

Close proximities between organelles have been described for decades. However, only recently a specific field dealing with organelle communication at membrane contact sites has gained wide acceptance, attracting scientists from multiple areas of cell biology. The diversity of approaches warrants a unified vocabulary for the field. Such definitions would facilitate laying the foundations of this field, streamlining communication and resolving semantic controversies. This opinion, written by a panel of experts in the field, aims to provide this burgeoning area with guidelines for the experimental definition and analysis of contact sites. It also includes suggestions on how to operationally and tractably measure and analyze them with the hope of ultimately facilitating knowledge production and dissemination within and outside the field of contact-site research.

## Abstract (web summary)

Given the recent growing interest in interorganelle membrane contact sites, the field will benefit from clear rules to define and study them. In this Perspective, a panel of experts aims to provide this growing field with guidelines for experimental definition and analysis.

## Introduction

In Eukaryotes, intracellular membranes delimit organelles that have distinct biochemical functions. While for decades the organelle field was governed by studies aimed at identifying the unique characteristics of each compartment, the last years have seen a revolution in the field as more focus is being placed on the interactions between the organelles and their role in maintaining cellular homeostasis.

Published examples of interactions between two distinct organelles appeared in the late 1950s^1,2^. However, the lack of a perceived physiological role made it hard to envision this as a general and functionally relevant phenomenon. The strong notion at the time was that the physical organization of the cytosol was performed solely by anchoring and movement on cytoskeletal elements. Moreover, it was believed that the transfer of small hydrophobic molecules between two organelles was catalyzed by freely diffusing cytosolic proteins, and that soluble metabolites and second messengers travelled long distances^3^. Together these two views delayed the appreciation of the importance of membrane tethering between two organelles.

The field started to expand and gain momentum when examples of functional apposition became evident and these areas were termed membrane contact sites. For example, when the juxtaposition between the endoplasmic reticulum (ER) and mitochondria was identified as the site of phospholipid biosynthesis and transfer^4^, and several years later as the site of efficient Ca^2+^ transfer^5^. Similar roles were then ascribed to the ER–plasma membrane (PM) contact site^6,7^ and to ER–Golgi contacts^8^. The discovery of the nuclear vacuolar junction (NVJ) and its role in piecemeal microautophagy of the nucleus^9,10^, and later the assignment of a role for the ER–PM contact in autophagy regulation^11^ showed that the role of contacts could be diverse. In recent years more roles have been uncovered such as for the ER in regulating mitochondrial^12–14^ and endosomal^15^ fission in contact sites between these organelles^16^. Roles in controlling inheritance such as in the case of peroxisomes^17^ have also been described. Hence, it is rapidly becoming evident that organelles are highly interconnected and that there are multiple important functions for these physical associations at contact sites.

In recent years additional contacts are being described and studied. More importantly, the previous functional observations are now being coupled by an increasingly clearer molecular understanding as tethers, molecules that bring and maintain the two membranes into close proximity, are being identified (for several broad reviews see^18–20^). Moreover, the uncovered molecular determinants are becoming implicated in a variety of cellular and pathophysiological processes^21,22^ demonstrating the importance of contact sites in normal development and physiology.

In sum, a whole new field has emerged, devoted to the investigation of the molecular mechanisms, the cell biology, the physiological and pathological implications of contact sites. While it is now obvious that such appositions are central to the structure and function of any eukaryotic cell and that they are becoming center-stage in cell biology research, it is also clear that, like any nascent scientific field, terminology, and experimental approaches to define and measure processes are vaguely defined, leading to potential controversies and hampering development of knowledge. To overcome this issue, we decided to offer a lexicon and a set of experimental guidelines to the field.

## What is a membrane contact site?

Membrane contact sites are classically defined as areas of close apposition between the membranes of two organelles. There have been, to date, examples of both homotypic (between identical organelles) and heterotypic (between two different organelles or two different membrane types) contact sites. Heterotypic contacts that have been well-studied originally all involved the ER. For example, the ER–mitochondria, ER–PM, ER–Golgi, ER–peroxisomes and ER-lipid droplets (LDs) contacts. Lately, contacts that do not involve the ER are being discovered such as: LDs–peroxisomes, mitochondria–vacuoles/endosomes/lysosomes, mitochondria–PM, mitochondria–LDs, mitochondria–peroxisomes, and mitochondrial inner and outer membranes (for a review on all characterized heterotypic contact sites to date see^18^). In plants chloroplasts engage in contact sites with most other organelles^20^. Homotypic contact sites, that are not fusion intermediates, have been described between two peroxisomes^23–25^ or two LDs^26^ and potentially other multicopy organelles could also form them. Homotypic interactions between organelles that are intermediates for fusion, in our eyes, are characterized by different features, and operationally do not represent a contact site similar to the ones discussed here and hence will not be touched upon in this review. It should also be noted that membrane-less organelles can form contacts with membranous organelles: for example, inclusion bodies can interact with LDs^27^. However, since these contacts do not occur between two organelles bound by membranes they might be physiologically very different, and consequently we will also not discuss them here.

## Features of a contact site

We now propose a set of unifying characteristics, which we consider essential features of contact sites. We suggest that an organelle juxtaposition can be defined as a contact site if it is characterized by the following.

### Tethering

We define contact sites as a *tethered* proximity between two bi- or mono-layer (such as LD) membrane-bound organelles. Many manuscripts define a “top limit” to the distance between the two organelles that can still be defined as a contact site (usually in the range of 10–80 nm distance with many focusing on 30 nm). Obviously, the narrower the gap, the easier and more obvious it is to see a contact. However, it is not clear how the specific distance value has been defined as some contact sites have the capacity to be much larger (see below for example Num1 that can span over 300 nm^28^). We hence suggest that distance cannot be a sole measure and that simple juxta-positioning of organelles is not sufficient to be considered a contact site regardless of distance. What does define a contact site in all cases reported to date, is the presence of tethering forces that arise from protein–protein or protein–lipid interactions.

### Lack of fusion

Contacts, in our view, *do not include intermediates* of an active, SNARE mediated or independent, fusion process. Fusion intermediates have been in the past referred to as “docking” events and this nomenclature, if kept, can help keep the two processes distinct. Limited vesicular trafficking between apposed organelles may, however, exist, but would follow established mechanisms and terminology.

### Specific function

Contacts *must fulfill a specific function*. Because the majority of contacts were initially described with the ER, this highlighted the role of contact sites in lipid and Ca^2+^ transfer^29^ and this dominated the view of the field for many years. It is now, however, becoming apparent, that virtually all organelles can contact each other and that the diversity of functions of these contacts is much wider. To date three types of functions have been suggested: (i) the specific bidirectional transport of molecules such as various ions, Ca^2+^, lipids, amino acids, and metals^30–32^. (ii) The transmission of signaling information or force important for remodeling activities, including regulation of organelle biogenesis, dynamics, inheritance, positioning, fission, and autophagy^13,14,16,17,33–37^. (iii) The positioning, *in trans*, of enzymes (such as the phosphatidylinositol (PI) phosphate phosphatase, Sac1; and the protein tyrosine phosphatase 1B, PTP1B^36,38,39^) so as to regulate their activity.

Since all contacts must have a function, this requires that they be regulated and hence that dysregulation of contacts should impact cell function and contacts should therefore be selected for by evolutionary pressure.

### Defined proteome/lipidome

Contacts should have a *functional protein and/or membrane composition* which is required for all of the above: tethering, function, regulation, and the maintenance of their architecture (see section below). The functions often benefit from concentration of specific proteins/lipids in the contact forming membranes, which creates a “quasi-synaptic” arrangement, where the organelles can locally and effectively cooperate with each other without altering the bulk cytosol^5,40^. In some cases, “moonlighting” may allow a protein to perform one function in the bulk of an organelle, but a contact-site-specific function when present there. At present, this may be harder to measure accurately. To date it has not been unequivocally shown that contacts have a unique lipidome but fractionation of ER–mitochondria contacts suggests that they are “raft-like” hence probably enriched in ceramides and sterols^41,42^.

It seems that the extent of time that a contact exists is flexible and dependent on its function, regulation, and cell type. Indeed, dynamic and transient contacts exist as well as stable ones that are maintained over long periods. Dynamic contacts have been described during, for example, pulsatile insulin secretion^43^, during ER–endosome contacts that control endosome motility^35^ following depletion of Ca^2+^ from ER stores^7,44^ or increase of intracellular Ca^2+^^45,46^. We suggest that period of existence is therefore not a defining characteristic of contacts. However, we recommend that the above four features all be experimentally characterized when a new type of contact is described.

## The protein composition of contact sites

**Fig. 1:**
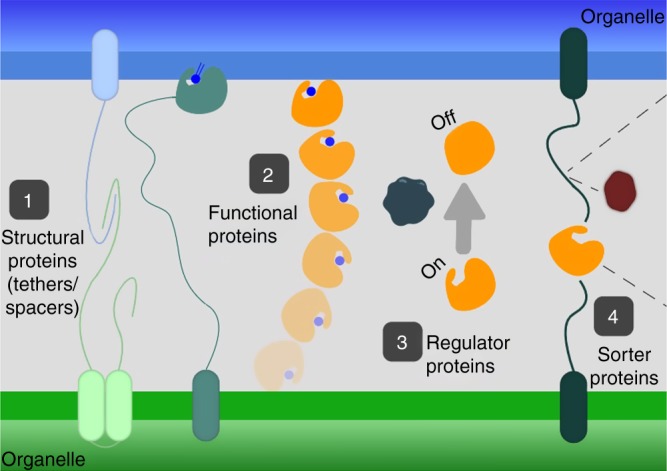
Graphical representation of the four types of proteins that should reside in contact sites. Importantly, many proteins can have multiple roles at a contact site

What type of proteins would reside in contacts? Originally contacts were thought to be populated by tethering proteins (tethers) that would establish/maintain the connection between the two membranes and by a second set of proteins that would fulfill functions specific to that particular contact site^29^. However, it is now appreciated that pairs of molecules functioning at contacts can also drive tethering and that the sum of the forces exerted by all these pairs ultimately tethers the two organelles^18^. This explains why, to date, most contacts have had multiple tethering molecules described and why eliminating a contact by deleting any singular tethering pair has proven impossible. However, each protein at a contact should have at least one role and we define four possible roles that should be found and enriched at contact sites (Fig. 1). It is important to note that proteins can, and often do, fall into more than one class and that current classification is based on the contact-site proteins known to date and additional classes should be added as they are discovered.

### Class 1: structural proteins

Structural proteins include tethers that hold the two organelles together and pillars/spacers that keep the two membranes at a defined distance and thus form the skeleton of a contact site as well as inhibit fusion. Spacers have not been well-studied to date. However, one example of active spacing is performed by extended-synaptotagmins (E-Syts) involved in ER–PM contacts in mammals^45^. As revealed by cryo-electron tomography (ET), the intermembrane distance between bilayers tethered by E-Syts is ~20 nm, which is exactly the length of the cytosolic region of these proteins^47^.

The number of studied tethers, however, is much larger and is increasing rapidly. Tethers often possess double targeting determinants to bind and bridge the two membranes. Tethers are often directly anchored to one of the two bilayers through a transmembrane domain or by a lipid modification (such as Vac8 undergoing palmitoylation^48^) and interact with proteins and/or lipids on the partner membrane through a second domain (i.e., E-Syt1/2/3, ORP5/8, LAMs, and Num1)^45,49–50^. Interaction with other proteins can also be homotypic^51–53^, but most tethers described to date are built on heterotypic interactions between two proteins on the opposing membranes. Some tethers like the VAPs have multiple interaction partners on the opposing membrane^54,55^. Generally, tethering pairs are structurally characterized by a protein domain composition that, ultimately, allows them to span the contact-site width over considerable distances (for example, up to 325 nm, in the case of Num1^56^).

To date it is thought that only very few proteins are pure tethers, with no other function. Such pure tether candidates include the yeast Nvj1, Junctophilin, and Num1^9,49,57^. However, even for these “pure” tethers, an additional function may yet be described. For all other tethering pairs an additional function at the contact site has already been characterized.

The presence of multiple tethering pairs ensures maintenance of contact, with some local variation depending on local enrichment of components, under a diversity of functional conditions. Recently, even contact sites that for years were considered to be held by a single tethering pair, such as the NVJ, have new tethering proteins ascribed to them such as the tether Mdm1^58^. Indeed, to abolish contact sites has proven impossible to date—with some contact sites requiring the ablation of six different proteins (for example, in the ER–PM contact: *∆ist2, ∆tcb1/2/3, ∆scs2*, and *∆scs22*) to see a dramatic reduction in contact (and even then ER–PM contact is not ablated completely)^66^. It seems that for the major contact sites complete ablation is not viable, but this has to be better studied to be confirmed.

Importantly, to date contact sites have been defined by the two organelles that they bring into proximity. However, recent reports suggest that in certain cases, such as mitochondria–vacuole^59^ and ER–PM^60^ contacts, different sets of tethers can form distinct sets of contact sites. Hence, in the future use of an umbrella term such as “the ER–PM” contact site may not be useful anymore and contact sites would be defined by both their opposing membranes and their component molecules or their specific functions^61^.

### Class 2: Functional proteins

Since each contact site must have a function, proteins that facilitate that function must reside in and be enriched at contacts. Such functional proteins can perform ion, protein, lipid, or metabolite exchange, for instance ion channels and pumps, lipid transfer proteins, or metabolite channels/transporters. It seems that in most cases described to date functional molecules also exert tethering capacity—for example, Lam6^50^ has a role in sterol transfer between mitochondria and ER membranes^128^, and itself also exerts a tethering role^62^. Similarly three proteins out of the ERMES complex that tethers the ER and mitochondria in yeast have lipid transfer domains^63^ as does PDZD8 that performs a similar role in mammals^64^, and the tricalbins (in yeast)^65–67^ or extended synaptotagmins (in mammals)^68^. Recently the Kv2.1 potassium channel has been shown to also have a role in tethering the ER to the PM^54^. Importantly, any functional protein that is proposed to also be a tether can be tested by re-expression of a version with mutation of the functional domain. Where this has not been done, even if partners on the opposing membrane exist, a role in tethering has to be taken as untested.

### Class 3: sorter/recruitment proteins

Sorting and recruitment proteins work to define the contact-site proteome and lipidome. This can be done by active recruitment of proteins into the contact site or by repelling non-residents of contact sites. Sorting can occur by direct binding to proteins or indirectly by either altering lipids (to define curvature and charge) or the proteins themselves (such as adding post-translational modifications). For example, it has been suggested that the mitochondria–ER contact site has a unique lipid composition relative to either membrane^69,70^ and that palmitoylation serves as a signal to enrich proteins at these unique membrane patches^71^. Some of the sorter proteins would work *in trans*, serving to maintain the membrane properties on the partnering organelles. Proteins in this group may also recognize lipid properties to enrich tethers or functional/regulatory proteins to contacts. Examples for these are the ER proteins Rab32 and phosphofurin acidic cluster sorting protein 2 (PACS-2), which regulate the intra-organellar distribution of contact-site proteins^72^. This group also includes PI transfer proteins, as well as PI kinases and phosphatases that are often enriched in contact sites^73,74^, since they could in theory define a unique lipidome for these areas.

### Class 4: regulator proteins

These include proteins that regulate the extent of the contact site itself as well as the function of the active proteins in the contacts. For example, phosphorylation of the tether holding the mitochondria–vacuole contact site controls tethering^75^. Another example is p53 that changes the redox state of Ca2^+^ handling proteins, thus altering ER–mitochondria tethering^76^.

Overall, the characterization of contact-site proteins has just begun and will likely continue to result in a plethora of mechanisms and players in the foreseeable future. However, we strongly suggest that a variable combination of these protein classes shall characterize any newly identified contact site. Importantly, the division of proteins into categories is purely synthetic as it is clear that many proteins have several capacities in the same molecule such as tethers that are also lipid transporters or regulators. Indeed, proteins belonging to these classes have all been identified in the several available proteomic studies of mitochondria–ER contacts^77^. Hence, this categorization is meant purely to aid in ordering the current knowledge and for ease of communication.

## Maintenance of membrane identity at contact sites

The two membranes engaged in contacts are stabilized by multiple tethering/functional complexes and kept in proximity without fusion (contact sites with LDs have hemifusion stalks that are unique^78^), thus preserving membrane identities. We propose fusion is inhibited at contacts by either:

### Repulsion

When found at very high proximity, two membranes will repel each other, and the repelling force increases exponentially as the water molecules that hydrate the phospholipid heads are squeezed out. In all reported cases of membrane fusion, fusion proteins are required to destabilize the bilayers enough to provoke lipid bilayer mixing. Juxtaposed membranes at contact sites must therefore be devoid of fusogens. The mechanisms that hinder the entry of fusogens into the contact sites have not been well-studied and would be interesting topics for future research.

### Spacing

Fusion occurs once lipid bilayers have been forced into a proximity of 1–2 nm, whereas all reported contacts are not closer than 10 nm in distance. Such spacing at contacts may be actively mediated by distancing proteins such as spacers. Such dedicated spacers have not yet been described in detail. However, it may very well be that spacing is simply a result of the large population of proteins resident at contacts that would need to be cleared before fusion can occur.

In conclusion, we suggest that newly identified contact sites are characterized for the presence of proteins that might inhibit fusion. The characterization of potential spacers could contribute to our understanding of interorganellar proximity without fusion.

## Distant relatives—fusion and tethering

Despite the distinctions between these two defined processes, that we designate as mutually exclusive, structural similarities exist between proteins that operate at contact sites and at fusion sites, and analysis of these similarities could contribute to a better understanding of contact formation and maintenance. For example, Synaptotagmin (Syt)1 binding to the SNARE protein complex is an essential step for membrane fusion^79^. However, at physiological ion concentrations Syt1 does not bind to SNAREs, but just to PIP_2_^80^ and acts as a Ca^2+^-dependent membrane tether, rather than a fusion-mediating protein, similarly to Extended (E)-Syts. Interestingly, a nonfusogenic SNARE complex has been reported to bridge the ER and PM in neurons^81^ and a SNARE has been shown to regulate ER–mitochondria contact-site functions^82^. Due to their amphiphilic nature, SNARE motifs can also associate in an antiparallel configuration^83^. While the parallel alignment would lead to vesicle–membrane fusion, an antiparallel configuration would not. These finding support a novel additional function for SNARE proteins in stabilizing contacts beyond their well-established role in membrane fusion and in the future maybe more of these examples will be found.

## Experimental approaches to study contact sites

Contact sites were first observed in electron micrographs (EM) as early as the 1950s, but for several years they were mostly overlooked given their transient nature or variable abundance within different cell types. In addition, to this day it remains challenging to visualize contact sites or purify them and therefore ascertain that they are present, understand their functions and study how environmental and genetic perturbations affect them. Hence, any study wishing to characterize contact-site components or functions faces first the issue of unequivocally identifying the contact, following its function and monitoring its changes upon perturbations. Below are approaches so far employed to study contacts as well as our recommendations for reliable contact-site visualization (See also Table 1 for the summary of pros and cons for each method).

**Table 1:** Pros and cons of the various experimental approaches to study contact sites

Approach	Pros	Cons
Epifluorescence and confocal microscopy	∙Live cell compatible ∙Fluorescent markers of organelles can be readily obtained ∙Can be used to visualize contact-site residents ∙Microscopes are readily available ∙Amenable to high-content approaches	∙Resolution limit of ~250 nm in *xy* and 500–700 in *z* is far larger than the size of most contact sites ∙Fixation for immunofluorescence microscopy may introduce artifacts
Super-resolution microscopy	∙Increased resolution over general fluorescence microscopy techniques ∙Some methods are live cell compatible	∙Highly specialized microscopes and accompanying expertise required ∙Fixation is required for some methods and may introduce artifacts
FRET-based reporters	∙Live cell compatible ∙Extremely sensitive to the distance between membranes ∙Can be used to examine contact-site dynamics	∙Technically challenging ∙Careful controls and equimolar expression of the FRET pair are required ∙Requires special microscopy set-up
Irreversible split fluorescence probes	∙Live cell compatible ∙No pre-existing knowledge of the contact site is needed ∙Enables discovery of new contact sites ∙Can be used as synthetic tethers for rescue experiments ∙Compatible with high throughput screening	∙Irreversible binding can stabilize, alter and expand sites of contact ∙Contact-site dynamics cannot be studied
Reversible fluorescence probes	∙Live cell compatible ∙No pre-existing knowledge of the contact site is needed ∙Can be used to examine contact-site dynamics	∙Low-fluorescence intensity of probes can restrict their application
Transmission electron microscopy (TEM)	∙High-resolution imaging of contact-site ultrastructure within the context of a cell ∙Considered the gold standard for the study of contact-site architecture ∙Can be combined with immunostaining to verify bona fide contact-site residents	∙Most useful for abundant contact sites or those whose residents can be readily detected using immuno-EM or CLEM approaches ∙Low throughput ∙Fixation may introduce artifacts
Electron tomography (ET)	∙Provides high-resolution 3D reconstructions of contact-site ultrastructure ∙Fully hydrated and unstained environment reduces artifacts	∙Technically challenging ∙Requires specialized equipment ∙Full 3D reconstructions not possible due to limited tilt range of the sample holder
Scanning electron microscopy (SEM)	∙Enables high-resolution 3D imaging of large specimen volumes	∙Resolving power more limited compared to other EM techniques ∙Time-consuming and computationally-intensive postacquisition processing of large datasets ∙Fixation may introduce artifacts
Cell fractionation	∙Allows for the proteomic and lipidomic analysis of isolated contact sites ∙Enables biochemical characterization of contact-site residents as well as activity	∙Contacts must be able to withstand the fractionation procedure ∙Purity is rarely achieved and contamination by other membranes is common ∙Protocols for most contact sites have not yet been developed
Proximity labeling	∙Does not require pre-existing knowledge of the contact site ∙Can be used to determine the proteome of a contact site when combined with mass spectroscopy ∙Can be used to identify residents of dynamic/transient as well as stable contacts	∙Requires careful controls
Proximity ligation assays (PLA)	∙Can provide quantitative information on contact-site distance and extent of contact ∙Good sensitivity	∙Requires antibodies to the proteins of interest ∙Fixation may introduce artifacts ∙Careful controls are required as changes in PLA signal do not always reflect changes in contact-site extent

### Epifluorescence and confocal microscopy of organelle proximities

Perhaps the most commonly used approach to visualize contacts is confocal microscopy of cells expressing fluorescent proteins targeted to the two compartments of interest, or where markers of the subcellular structures of interest have been appropriately immunostained. This approach was introduced in a landmark paper identifying ER–mitochondria juxtaposition as sites of Ca^2+ ^transfer between the two organelles^5^. There, it was performed on live cells expressing GFP spectral variants targeted to the two organelles, coupled to fast imaging, deconvolution and 3D reconstruction of ER and mitochondria. This approach has been extended by using multi-spectral image acquisition to visualize six different organelles simultaneously and compute their areas of contact^84^.

While tagging two organelles each with a different color is fast, easy and amenable to adaptation to high-content approaches, it suffers from intrinsic limitations. First, most contact sites are smaller than the optical diffraction limit: on the *x–y* axis, resolution is limited to 250 nm; on the *z*-axis, point spread functions of microscopes limit resolution to approx. 500-700 nm. Second, chemical fixation and single-plane confocal microscopy can alter contacts and offer a partial representation of interactions that occur in three dimensions. Third, visual quantification of proximity measured in confocal microscopy experiments must be accompanied by indexes of pixel-by-pixel overlap like Pearson’s and Manders’ coefficients. In conclusion, while contact-site measurements based on confocal pseudo colocalization experiments are important, they are more conclusive when (i) accompanied by a second approach that is endowed with a resolution power amenable to detect distances in the range of contact sites; (ii) performed on live cells, using piezoelectric z-stepper or other similar approaches, to acquire the whole cellular volume in very short times; (iii) accompanied by careful experiments of reconstitution of organelle shape and number to exclude possible artifacts caused by variability in these traits.

### Epifluorescence and confocal microscopy of contact-site residents

A different approach to visualize contact sites is based on imaging of characterized contact-site residents. Such residents often have a unique punctate appearance specific to the interface and are therefore very useful for accurate representation of the interaction space. While this approach has been powerful in enabling live imaging of contact sites in various cell types, one must remember that the various residents may not reside in the entire contact site and that it may even be that different contacts exist between the same two organelles^60^. Hence, different patterns for a contact site between the same organelles might result from using different markers. For example, using different tethering molecules to visualize the ER–PM contact gives rise to visualization of domains that are shaped as “patches” (when using E-Syt2/3^45^) or “punctae” (when using ORP5/8^49,85^). In addition, such approaches are clearly limited to the analysis of a subset of contacts whose components have already been identified.

### Proximity ligation assays for quantifying organelle proximities

Proximity ligation assays (PLA) between two contact-site components on opposing membranes have been employed to extract quantitative information on the distance between two membranes or the extent of contact^86^. This can be very powerful to study alterations that occur in response to genetic or environmental perturbations. While this approach can indeed give a sense of membrane proximities, it also suffers from a number of limitations: first, reductions in PLA signals can be caused by changes in expression or localization of one of the PLA partners; second, it requires that the PLA partners are unequivocally localized at the contact site; third, it might not recognize changes in contact-site extent not accompanied by changes in proximity between the two proteins measured in the PLA. We, therefore, believe that PLA can be a powerful tool to corroborate protein–protein interaction in situ especially in *trans*, but we do not recommend PLA as the tool to measure contact extent. If used it should be very carefully controlled.

### FRET/split fluorescence reporters of contacts

Similarly, to PLA, fluorescence resonance energy transfer (FRET) and split fluorescence sensors can detect proximities between two membranes based on the effect of proximity on the fluorescence of the pair and can be used in live cells. Such probes can be broadly divided in three classes:

*FRET-based reporters:* The transfer of energy between two fluorophores has been used to visualize organelle proximities such as ER–mitochondria juxtaposition^87^. Importantly, FRET reporters are exquisitely sensitive to the distance between the membranes as energy transfer is inversely proportional to the sixth power of distance between the fluorophores, and since no dimerization occurs. By adding a rapamycin-induced dimerization domain, maximal FRET measurements can be obtained, thus enabling quantitative measurements of contact distance at any given time point and cross-sample comparison. The greatest shortcoming of this probe is the requirement for equimolar expression of the two FRET pairs (unless FRET is measured by fluorescence-lifetime imaging microscopy, which is not affected by the relative amount of the two fluorophores). A modified version of an ER–mitochondria FRET-based probe, overcame this complication by expressing the two fluorophores as a single mRNA with a self-cleavable TAV2a sequence between them^88^. By changing the targeting sequence of the individual fluorescent proteins, this approach can be adapted to measure any potential contact site. However, FRET measurements require proficient experimenters and dedicated equipment, thereby limiting the utilization of such probes.

*Irreversible split fluorescence probes*: Split fluorescence probes, such as split Venus or green fluorescent protein (GFP), are based on the appearance of a fluorescent signal when two nonfluorescent fragments targeted to the partner organelles bind each other once the juxtaposed membranes are in proximity^18,52,89–91^. Since it is now clear that a signal appears at the interface no matter which proteins on the two organelles are chosen to be tagged^18^, this approach does not require any pre-existing knowledge about a contact site and enables discovery of novel contact sites. However, these probes suffer from the fact that complementation of the two fragments is thermodynamically stable, leading to contact-site stabilization. In such approaches the dynamics of contact sites cannot be studied, and some contacts may become toxic under some conditions, when they cannot be eliminated. It is important to also remember that these approaches do not discriminate between close associations of organelles and tethered contacts and can cause synthetic expansion of the associations.

*Reversible or quasi-reversible fluorescent probes*: This method is similar to the above method of bimolecular complementation but depends on usage of protein fragments that have very low intrinsic affinity of the two halves of the fluorophore. So far, two types of such probes have been utilized for contact-site detection: ddGFP^88,92^, which is based on a reversibly dimerizing dsRED fluorescent protein genetically modified to emit in the green zone of the light spectrum (hence called GFP); and split infra-red reporters^52,93^. However, the intrinsically low fluorescence of these probes might restrict their application. Brighter versions will be required for increased usage.

Importantly, all above probes enable proximity measurements between two organellar membranes. While this can be highly important for detecting the presence of a contact site, it should be utilized carefully in measuring the effect of any single tether, functional molecule or spacer. To date, all contact sites described have more than a single tethering pair in them—hence loss of any one protein does not necessarily cause reduction in either the amount of contact or the distance between organelles. Moreover, increased distance between two organelles does not necessarily mean loss of contact and could also represent the formation of alternate, wider, contact areas by an alternate tethering mechanism as a compensation.

### Super-resolution and atomic force microscopy

Since contacts are below the diffraction limit of light microscopy, super-resolution^94^, or atomic force microscopy^95^ can be powerful tools to accurately visualize them and study the localization and the distribution of the proteins that reside within these structures. Super-resolution methods that can be used include structured illumination microscopy, stimulated emission depletion (STED) microscopy and single molecule localization microscopy, however, all such techniques require highly dedicated microscopes and technical expertise, and despite the ongoing development of sub-diffraction microscopy techniques, their application to contact-site analysis is still limited.

### Transmission electron microscopy

Transmission electron microscopy (TEM) is an atomic-resolution microscopy system that provides static, high-resolution, ultrastructure information on contact sites within the cellular context^45,85,96^, which very often cannot be explored with other experimental approaches. Hence, TEM is often considered the “gold standard” for the study of the fine architecture of contact sites and to reveal their morphological and functional diversity. However, this approach is often only useful for either highly abundant contact -sites (rare contact sites will not be spotted in EM fields), or those that have very good marker proteins that can be used for immunodetection^75,97,98^ or correlative light electron microscopy (CLEM) approaches^99^. The latter two approaches can also both be used to pinpoint a protein specifically at a contact site and is therefore considered the best proof of it being a resident protein.

Unfortunately, TEM is a very low-throughput technique, affected by the commonly used chemical fixation procedures, limited to the analysis of a single plane, and therefore not suitable to measure the extent of contact in cells unless multiple parameters are taken into consideration (surface of the organelles of interest, extent of the contact, intermembrane distance at the contact etc…) and computed into one index that is applied in extensive morphometric experiments^100^. We therefore recommend that TEM be used to provide qualitative and quantitative features of contact sites but not to study occurrence and changes in the extent, unless rigorous morphometric analyses are included.

### Electron tomography (ET)

An exciting “spin-off” of conventional TEM is ET. Conventional and cryo-ET offer the advantage of generating three-dimensional (3D) reconstructions of a cellular structure in a fully hydrated and unstained environment, providing more exhaustive and complete insights into its organization and possible functions. In ET multiple images are captured as the sample is tilted along an axis. The images are then aligned and merged using computational techniques to reconstruct a 3D picture, or tomogram. ET can be combined with immunostaining, allowing localization of proteins in the 3D reconstructions^101^. ET has been successfully employed to study contact sites such as the ER–mitochondria^53,96^ or ER–PM^47^ as well as to generally map contact sites in small structures such as axons^102^. ET is the most reliable approach to validate the presence of linkers at the contacts^103,104^.

However, ET often requires serial sectioning to reconstruct the 3D model of the entire structure, and the combination of such laborious approaches could be technically challenging. Also, it should be considered that the 3D reconstructions created from ET tilt series of images are not complete representations. This is due to the limited tilt range of the microscope holder in ET that leaves the 3D reconstruction with regions empty of information appearing as undefined cone shaped areas (so-called “missing wedge”). Nonetheless, combined with advances in cryo-ET sample preparation, such as the introduction of focused ion beam milling to thin samples to ideal thicknesses for imaging, the potential for cryo-ET imaging is constantly increasing.

### Scanning electron microscopy

Volume EM techniques based on scanning electron microscopy (SEM) enable high-resolution 3D imaging of large specimen volumes. 3D-SEM techniques overcome the artifact of the “missing wedge” in ET, and are therefore a potent tool for gaining insight into the 3D morphology of contacts^105^. However, despite recent improvements, 3D-SEM resolving power is still limited and requires extended research time and computer power for processing the large amount of datasets produced.

### Cell fractionation

The biochemical characterization of contact sites occurred contemporaneously with their discovery by EM. Commonly, protocols for isolating membrane contacts utilized subcellular fractionation followed by sucrose gradient centrifugation. Any successful isolation of heterotypic contacts must consider that these have features of two organelles or maybe of neither. Moreover, purification of such contact regions can be challenging as they need to resist dilution and cell fractionation. Additionally, during the lengthy fractionation procedure, potential protein modifications and interactions (such as phosphorylation or dimerization) might be reversed, which could destabilize or alter contacts.

Most of the early protocols that biochemically detected interorganellar contacts dealt with areas of strong association between ER and mitochondria. As early as the 1950s, it was observed that liver mitochondria preparations obtained from sucrose fractionation were “always contaminated with ER”^106^. The characterization of lipid-synthesizing membranes in the early 1970s assigned mitochondrial lipid synthesis to these contacts, although this was not formally recognized^107–109^. Following this insight, attempts to subfractionate domains of the ER led to further insight about this contact such as that it comprised only a specific portion of the rough ER membrane^110–112^. The real breakthough came with the identification of specific enzymes enriched in these domains, cementing the understanding that these were unique sites of lipid transfer between the two organelles^4,113–115^. These sites, from thereon termed mitochondria-associated membranes, became the first functional characterization of a contact-site function.

Similar to ER associations with mitochondria, the isolation of the PM via sucrose gradients from reticulocytes led to the identification of ER tethered to the PM^116^, but the significance of this finding, in particular for Ca^2+^ entry, was initially overlooked. PM-associated membranes were also shown to have unique lipid biosyntehtic capacities^6^.

The biochemical isolation of ER–peroxisome contacts (EPCONS)^117–119^ takes advantage of the sustained attachment of ER tubules to peroxisomes under conditions that separate mitochondria from peroxisomes. Moreover, they have enabled proteomic analysis of these contacts^117^.

Hence, biochemical fractionation assays for contact sites are important tools for studying functional aspects of the contacts as well as identifying their unique lipid composition and resident proteins. Unfortunately, such protocols have not yet been developed for all contact sites.

### Proximity labeling

A new way to define contact-site proteins is by using biotinylation approaches. APEX^120^ has been used to identify new contact-site proteins^121^. At the heart of this methodology lies an engineered version of ascorbate peroxidase (APEX), which can catalyze the oxidation of biotin phenol. This radical version of phenol is both short lived (<1 ms), has a small labeling radius (<20 nm), and can covalently react with electron-rich amino acids (Tyr, Trp, His, and Cys). Thus, by localizing the APEX protein to a contact site and then shortly exposing the cell to hydrogen peroxide and biotin phenol, all proteins within this compartment will be modified by biotin. Biontinylated proteins can then be easily extracted using the well-established streptavidin system, and identified via mass spectrometry. If no proteins are known for a specific contact it is possible to map the entire proteome of the outer membrane of two organelles and then look for overlapping proteins which may be residents of the contact sites^122^. APEX has also been used alongside biochemical fractionation to reach a better purity of contact residents^121^. In addition, a split APEX^123^ has recently been created, enabling each half to be expressed on one organelle and complementation to occur only at contact sites. Similarly, a nonspecific biotin ligase such as the BirA* can be used to biotinylate proximal proteins in a method called BioID^124,125^. A split-BioID has been described^126^ which, again, can be used to study contact sites by labeling two opposing membranes and retrieving biotinylation activity only at the areas of interface.

### Functional studies and genetics

Since each contact site has its unique function, different approaches will be required to measure the functionality of a contact. For example, a great deal of attention has been given to transfer of lipids^127^ and Ca^2+^^53^ at ER–mitochondria or at ER–PM contacts. Such approaches should be developed to appraise if changes in contact extent or composition affects cellular physiology. Appropriate functional analyses need to be performed in combination with the imaging approaches detailed above, to draw conclusions on whether contact sites are present and/or changed in the studied system.

Care should be taken not to confuse a lack of effect on any singular function as an indication that a protein is not a tether or a functional contact-site protein. This is because deletion of a tether is often backed up by many other tethering molecules and its effect will therefore not necessarily be measurable if a function that it does not carry out is measured (for example, deletion of a lipid-binding tether which probably functions in lipid transfer and measurement of the effect of Ca^2+^ transfer).

Conversely, many tethers have functions outside of the contact site and thus their loss-of-function phenotype may not be due to their contact-site role. To give just one example, many of the mitochondrial contact-site proteins in yeast (Mdm10, Tom70 and Tom40^59,62,128,129^) function in both tethering as well as mitochondrial protein translocation. In such cases, it is necessary to generate separation-of-function alleles to inactivate just contact sites while maintaining the other function.

## Conclusions and future directions

In summary, this opinion offers a compendium of techniques and guidelines to construct appropriate experiments for the reliable  definition of contact sites and their modulation by established or newly discovered structural components. Our take-home advice to the field and to the colleagues interested in contact sites is to always combine biochemical, fluorescent, and EM approaches. Visualization of contact sites must be complemented with measurements of function such as: movement, fission, inheritance, ion exchange^53^, or lipid transfer^127^.

Many new and exciting questions remain to be answered in the field of contact sites—are there more contact sites that have not yet been described? Are there different varieties of contact sites between similar organelles? What are all the molecular tethers, spacers, and other contact residents? How is the distribution of signalling proteins determined between contact sites and non-junctional areas? And when these proteins are multi-subunit complexes (such as the mitochondrial calcium uniporter, mcu), does their molecular composition differ? What is the repertoire of functions carried out at contact sites? How does the loss of each contact affect cellular physiology and organismal function? How are contact sites regulated and co-regulated to maintain cellular homeostasis? And, given the apparent inability to completely deconstruct contact sites, what mechanisms exist that compensate malfunctions?

As it is becoming clear that contact sites affect a variety of diseases^21,130,131^, interest in these structures will only increase. We anticipate that in the next decade the above questions will start to be addressed, calling for extended guidelines that help define good practices in these additional areas of contact-site research.
